# Identifying the molecular targets and mechanisms of xuebijing injection for the treatment of COVID-19 via network pharmacology and molecular docking

**DOI:** 10.1080/21655979.2021.1933301

**Published:** 2021-06-02

**Authors:** Zhao Tianyu, Guan Liying

**Affiliations:** a Department of Pharmacology, College of Basic Medical Sciences, Jilin University, Changchun City, Jilin Province, People’s Republic of China; b Department of Pharmacy, China-Japan Union Hospital, Jilin University; Changchun City, Jilin Province, People’s Republic of China

**Keywords:** AKT1, covid-19, molecular docking, network pharmacology, xuebijing injection

## Abstract

Xuebijing Injection have been found to improve the clinical symptoms of COVID-19 and alleviate disease severity, but the mechanisms are currently unclear. This study aimed to investigate the potential molecular targets and mechanisms of the Xuebijing injection in treating COVID-19 via network pharmacology and molecular docking analysis. The main active ingredients and therapeutic targets of the Xuebijing injection, and the pathogenic targets of COVID-19 were screened using the TCMSP, UniProt, and GeneCard databases. According to the ‘Drug-Ingredients-Targets-Disease’ network built by STRING and Cytoscape, AKT1 was identified as the core target, and baicalein, luteolin, and quercetin were identified as the active ingredients of the Xuebijing injection in connection with AKT1. R language was used for enrichment analysis that predict the mechanisms by which the Xuebijing injection may inhibit lipopolysaccharide-mediated inflammatory response, modulate NOS activity, and regulate the TNF signal pathway by affecting the role of AKT1. Based on the results of network pharmacology, a molecular docking was performed with AKT1 and the three active ingredients, the results indicated that all three active ingredients could stably bind with AKT1. These findings identify potential molecular mechanisms by which Xuebijing Injection inhibit COVID-19 by acting on AKT1.

## GRAPHICAL ABSTRACT

**Figure:**
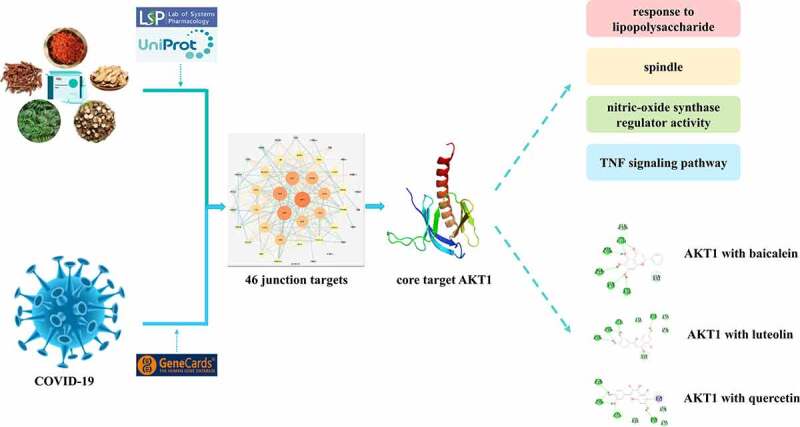

## 1. Introduction

Corona Virus Disease 2019 (COVID-19) is an infectious disease caused by severe acute respiratory syndrome coronavirus 2 (SARS-CoV-2). It was first identified in December 2019 in Wuhan, Hubei, China, and has since spread around the world. The WHO has declared that the COVID-19 outbreak constitutes a Public Health Emergency of International Concern (PHEIC). This disease can be clinically classified as mild, severe, or critical. Fever, dry cough, and fatigue are the main manifestations, and patients classified as severe can rapidly progress to acute respiratory distress syndrome (ARDS) and multiple organ failure (MOF), amongst other conditions. Unfortunately, at present, there is no cure officially approved for this disease, creating a formidable challenge in its treatment, prognosis, and control. Traditional Chinese medicines (TCM), that are characterized as being anti-viral and affecting multiple pathways and targets, have been proven to be significantly effective in treating COVID-19.

The Xuebijing (XBJ) injection is a traditional Chinese medicine that is based on the XueFuZhuYu Decoction (XFZYD). Its main herbal components are Carthami Flos, Paeoniae Radix Rubra, Chuanxiong Rhizoma, Salviae Miltiorrhizae Radix et Rhizoma, and Angelicae Sinensis Radix, and it can have several effects, such as anti-inflammation, anti-oxidation, immune regulation, improving blood stasis, anti-endotoxin, and anti-shock. Previously, it has been used to treat patients with diseases like severe pneumonia and sepsis. In 2019, the *National Drug Catalog for Basic Medical Insurance, Work-related Injury Insurance and Maternity Insurance* stated that XBJ injection should be used in the first aid treatment of critically ill patients. Furthermore, the *Diagnosis and Treatment Protocol for COVID-19* (Trial Versions 4, 5, 6, 7, 8) states that it should be used to treat severe and critically ill patients with COVID-19. However, its mechanisms in the treatment of COVID-19 have not yet been elucidated.

Highly pathogenic viruses should be handled under strict laboratory conditions and in compliance with specific biosafety procedures, this study has attempted to investigate the possible molecular mechanisms of XBJ injection when treating COVID-19 via computer simulation based on network pharmacology and molecular docking analyses; thereby avoiding potential laboratory biosafety hazards and improving the efficiency of research into infectious disease outbreaks and modern Chinese medicines. Recently, network pharmacology-based drug repositioning has become increasingly important for the research and development of drugs. Network pharmacology is an application tool based on systems biology theory and network analysis statistics, that conducts network analysis for specific biological systems, and designs multi-target drug molecules by selecting specific signal nodes and comparing signal pathways. Zhou et al. [1] identified 16 candidate drugs and 3 potential drug combinations for the treatment of human coronaviruses (HCoVs) through network predictions using over 2000 FDA-approved drugs and systems pharmacology and network pharmacology analyses. These results provide guidance for drug repositioning in the prevention and control of COVID-19. Molecular docking is an important computer-aided drug design method used to study receptor-ligand interactions such as the conformational space, binding energy, and chemical environment via stoichiometry. It has been widely used to elucidate and predict the therapeutic effects, as well as the underlying mechanisms of drugs. In recent years, molecular docking has developed into a common and preferred technique when reseraching Chinese medicines and has exhibited proven efficiency when screening for active ingredients [2,3].

This study has improved upon the network pharmacology and molecular docking anlaysis methods, and determined how to identify the core target in the gene network using the maximum Degree value, which indicates the role of the nodes in network pharmacology. The cluster Profiler package in R language was then used to screen the latest Gene Ontology and KEGG pathways for the core target. The network pharmacology results were verified using protein-small molecule docking to simulate the binding between active ingredients and the core target. These analyses were used to predict the active ingredients, targets, and signaling pathways, provide a molecular basis to investigate the mechanisms by which the COVID-19 inflammatory stress response is inhibited by the XBJ injection, as well as enhance the available theoretical support for the wider application of this drug in the treatment of COVID-19.

## 2. Materials and methods

### 2.1 Main active ingredients, potential targets, and network building for the XBJ injection

#### 2.1.1 Screening of the main active ingredients and potential targets for the XBJ injection

The main active ingredients were screened from the 5 main herbal components of the XBJ injection, including Carthami Flos, Paeoniae Radix Rubra, Chuanxiong Rhizoma, Salviae Miltiorrhizae Radix et Rhizoma and Angelicae Sinensis Radix using the Traditional Chinese Medicine Systems Pharmacology Database (TCMSP [4],http://tcmspw.com/tcmsp.php) according to oral bioavailability (OB) ≥ 30% and drug likeness (DL) ≥ 0.18. The drug targets of the main active ingredients were then screened through the Targets information of TCMSP. Finally, gene symbol conversion was performed for the targets screened from the TCMSP according to UniProt [5] (https://www.uniprot.org/). Additional information was added according to the literature, and 206 targets were obtained.

#### 2.1.2 Construction of the ingredient-target network for the XBJ injection

Cytoscape 3.7.2 [6] was used to import the main active ingredients and targets of the XBJ injection, and the active ingredients were numbered to build the ingredient-target network.

### 2.2 Screening of COVID-19 targets

In Genecards (https://www.genecards.org/) [7], coronavirus disease 2019, coronavirus pneumonia, coronavirus, and novel coronavirus 2019 were used as the keywords for retrieval respectively, and the results were exported in Excel. Then 277 potential targets for COVID-19 were screened for score ≥1 and the information was added according to the literature.

### 2.3 Analysis of the XBJ targets when treating COVID-19

#### 2.3.1 Construction of the protein-protein interaction (PPI) network

Junction targets for the XBJ injection and COVID-19 were imported into the STRING database (https://string-db.org/cgi/input.pl) [8], and the relevant parameters were set as follows:

1. Basic Settings: ① Network type: full network (the edges indicating both functional and physical protein associations); ② meaning of network edges: evidence (line color indicates the type of interaction evidence); ③ active interaction sources: Textmining, Experiments, Databases, Co-expression, Neighborhood, Gene Fusion, and Co-occurrence; ④ minimum required interaction score: medium confidence (0.400); ⑤ max number of interactors to show: 1st shell: none/query proteins only, 2nd shell: none.
2. Advanced Settings: ① network display mode: interactive svg; ② network display options: hide disconnected nodes in the network.

The PPI network for the junction targets for the XBJ injection and COVID-19 was constructed using the STRING database, and the results were exported to a TSV(Tab-separated values) file for further analysis using Cytoscape 3.7.2 (① for analysis, click Tools > Network Analyzer > Network Analysis > Analyze Network > Treat the network as undirected; ② for mapping, click Tools > Network Analyzer > Network Analysis > Generate Style from Statistics) to optimize the PPI network and screen for the core target with the maximum Degree value.

2.3.2 GO enrichment analysis and KEGG pathway analysis for the core target for the XBJ injection and COVID-19

Gene Ontology (GO) includes three aspects: biological process (BP), cellular component (CC) and molecular function (MF). GO enrichment analysis and Kyoto Encyclopedia of Genes and Genomes (KEGG) pathway analysis were conducted for the core target for the XBJ injection and COVID-19 using R (version 3.6.1) [9] and the clusterProfiler [10] package, respectively. In addition, Cytoscape 3.7.2 was used to plot the results.

### 2.4 Verification of the molecular docking for the results of network pharmacology

According to the network pharmacology results, AKT1 was identified as the core target of the XBJ injection when treating COVID-19, and the active ingredients of the XBJ injection, baicalein, luteolin, and quercetin were all connected with the core target AKT1. Consequently, further molecular docking verification was carried out based on these results.

RCSB PDB databases were used to download the 3D structure PDB file of the core target AKT1 (PDB ID: 1UNP), and it was then imported into the Discovery Studio 2019 Client (DS) to remove solvent molecules and ligands. Solvents were removed from the structure, while polar hydrogen atoms were added to the 1UNP by clicking ‘Add Polar’, and a forcefield was applied to the 1UNP by selecting ‘Apply Forcefield’. The PubChem database was used to obtain the SDF files of the active ingredients for the core target AKT1 as small molecules, which were imported into the DS. Apply Forcefield was used to give the force field to the small molecules. Finally, the CDOCKER (Dock Ligands) was used for protein-small molecule docking, by adjusting the Top Hits-Pose Cluster Radius to 0.5, and the other parameters were their default values. CDOKER [11] is a high-accuracy molecular docking method based on the CHARMm force field. With this method, high-temperature kinetics are used to search for the flexible conformation of the ligand molecules. The lower the CDOCKER interaction energy is, the more stable the conformation of the ligand binding to the receptor is thought to be. Furthermore, ≤−5.0 kcal·mol^−1^ indicates that the protein and small molecule can bind, and ≤−7.0 kcal·mol^−1^ indicates a strong binding ability.

## 3. Results

This study aimed to investigate the molecular targets and mechanisms of the Xuebijing injection when used to treat COVID-19 using network pharmacology and molecular docking analyses. According to the ‘Drug-Ingredients-Targets-Disease’ network, AKT1 was identified as the core target, and baicalein, luteolin, and quercetin were identified as the active ingredients of the Xuebijing injection that were associated with AKT1. The results of the GO enrichment analysis showed that response to lipopolysaccharide was the most significant biological process in which AKT1 was involved, the cellular component was spindle, and its molecular function involoved nitric-oxide synthase regulator activity. The results of the KEGG pathway analysis showed that AKT1 was involved in the treatment of COVID-19, mainly by regulating TNF and other signaling pathways. Based on the results of the network pharmacology, molecular docking analysis was performed with AKT1 and the three active ingredients, and the results indicated that all three active ingredients could bind stably with AKT1.

### 3.1 Main active ingredients, potential targets and network building for the XBJ injection

#### 3.1.1 Screening of the main active ingredients and potential targets

The 5 herbal components were retrieved using TCMSP. According to the screening requirements of an OB ≥ 30% and DL ≥ 0.18, we identified 44 active ingredients, including 9 from Carthami Flos, 8 from Paeoniae Radix Rubra, 6 from Chuanxiong Rhizoma, 19 from Salviae Miltiorrhizae Radix et Rhizoma, and 2 from Angelicae Sinensis Radix. MOL000449-Stigmasterol and MOL000358-beta-sitosterol were the common active ingredients mapping to Carthami Flos, Paeoniae Radix Rubra, and Angelicae Sinensis Radix. MOL002714- baicalein was the common active ingredient mapping by Carthami Flos and Paeoniae Radix Rubra, while MOL000006-luteolin was the common active ingredient mapping to Carthami Flos and Salviae Miltiorrhizae Radix et Rhizoma. Following the removal of the duplicates, a total of 38 non-repeated active ingredients were identified. In addition, 206 non-repeated drug targets were obtained from the TCMSP, according to the active ingredients mentioned above.

#### 3.1.2 Construction of the ingredient-target network

**Figure 1.**
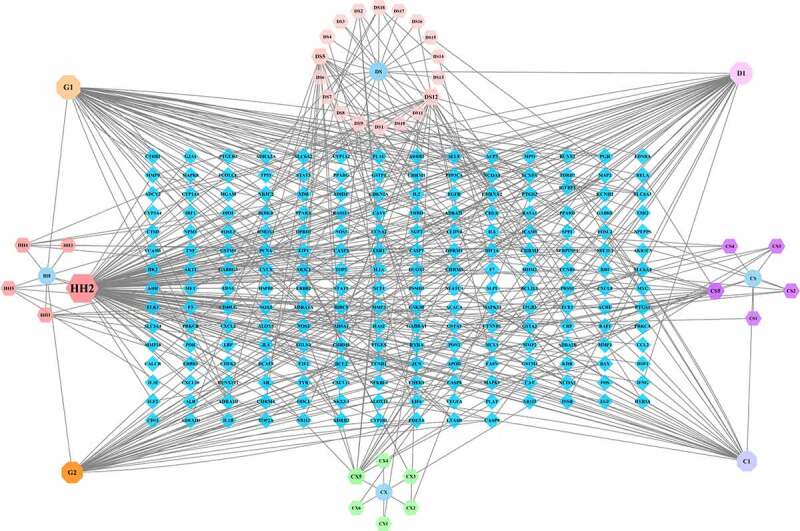
Ingredient-target network for the XBJ injection. HH denotes the active ingredient of Carthami Flos; CS denotes the active ingredient of Paeoniae Radix Rubra; CX denotes the active ingredient of Chuanxiong Rhizoma; DS denotes the active ingredient of Salviae Miltiorrhizae Radix et Rhizoma; G1 and G2 denote the active ingredients of Carthami Flos, Paeoniae Radix Rubra and Angelicae Sinensis Radix; C1 denotes the common active ingredient of Carthami Flos and Paeoniae Radix Rubra; D1 denotes the common active ingredient of Carthami Flos and Salviae Miltiorrhizae Radix et Rhizoma; blue diamonds represent the targets

The ingredient-target network for the XBJ injection was constructed using Cytoscape 3.7.2 and included 38 non-repeated active ingredients and 206 non-repeated potential targets (see Table 1 and Figure 1). The core targets in the ingredient-target network, including PTGS1, PTGS2, NCOA2, KCNH2, SCN5A, ADRB2, RXRA, PGR, NR3C2, NCOA1, and PDE3A, were screened according to Degree ≥ 5.

**Table 1.** XBJ injection Herbal Components- Active Ingredients- ID

Herbal Components	Active Ingredients	ID
Carthami Flos	Stigmasterol	G1
Paeoniae Radix Rubra		
Angelicae Sinensis Radix		
Carthami Flos	beta-sitosterol	G2
Paeoniae Radix Rubra		
Angelicae Sinensis Radix		
Carthami Flos	Baicalein	C1
Paeoniae Radix Rubra		
Carthami Flos	Luteolin	D1
Salviae Miltiorrhizae Radix et Rhizoma		
Carthami Flos	6-Hydroxykaempferol	HH1
	Quercetin	HH2
	Lignan	HH3
	Kaempferol	HH4
	beta-carotene	HH5
Paeoniae Radix Rubra	Paeoniflorgenone	CS1
	(2 R,3 R)-4-methoxyl-distylin	CS2
	(+)-catechin	CS3
	Paeoniflorin	CS4
	ellagic acid	CS5
Chuanxiong Rhizoma	FA	CX1
	Perlolyrine	CX2
	Wallichilide	CX3
	Mandenol	CX4
	Myricanone	CX5
	Sitosterol	CX6
Salviae Miltiorrhizae Radix et Rhizoma	przewalskin b	DS1
	(2 R)-3-(3,4-dihydroxyphenyl)-2-[(Z)-3-(3,4-dihydroxyphenyl)acryloyl]oxy-propionic acid	DS2
	(6S)-6-hydroxy-1-methyl-6-methylol-8,9-dihydro-7 H-naphtho[8,7-g]benzofuran-10,11-quinone	DS3
	Formyltanshinone	DS4
	Epidanshenspiroketallactone	DS5
	prolithospermic acid	DS6
	2-(4-hydroxy-3-methoxyphenyl)-5-(3-hydroxypropyl)-7-methoxy-3-benzofurancarboxaldehyde	DS7
	Danshenol A	DS8
	Cryptotanshinone	DS9
	Danshenspiroketallactone	DS10
	Isotanshinone II	DS11
	tanshinone iia	DS12
	dihydrotanshinonel	DS13
	salvianolic acid j	DS14
	2-isopropyl-8-methylphenanthrene-3,4-dione	DS15
	Miltirone	DS16
	4-methylenemiltirone	DS17
	Salviolone	DS18

### 3.2 Screening of COVID-19 targets

GeneCards was used to identify 277 targets of COVID-19, and STRING and Cytoscape were used to screen out the free targets. These targets, such as TNF, IL6, GAPDH, TP53, and IL10, were then arranged in order of degree (see Table 2).

**Table 2.** Targets of COVID-19

Degree	Gene Symbol	Degree	Gene Symbol	Degree	Gene Symbol	Degree	Gene Symbol	Degree	Gene Symbol
154	TNF	55	TGFB1	33	TXN	20	BAG3	10	CSNK2A2
153	IL6	54	CXCL9	33	PML	20	PPIA	10	HAVCR1
150	GAPDH	53	RPS27A	33	HLA-A	20	CTSL	10	PRSS3P2
139	TP53	52	HSPA5	33	IL16	19	PSMC1	10	BMP6
118	IL10	52	LCK	32	SUMO1	19	USP7	9	EIF2AK4
117	ALB	52	STAT2	32	VCP	19	TF	9	LMAN1
111	IL2	51	PIK3CA	32	NPM1	18	BAK1	8	POLD1
110	MAPK3	51	IFIH1	32	ANXA2	18	G6PD	8	TRIM56
109	CASP3	51	CCL4	32	LCN2	18	IFITM2	8	SFTPD
107	CXCL8	50	SMAD3	31	EEF1A1	18	IRAK3	8	FGL2
106	IFNG	50	NOS2	31	CCL7	18	PPP1R15A	7	RCHY1
105	EGFR	49	BECN1	30	CDK4	18	FURIN	7	PI4KB
105	IL4	49	ITGB1	30	DUSP1	18	CD79A	7	KPNA4
104	MAPK8	49	UBB	30	EIF2AK2	18	ADA	7	NLRP12
102	MAPK1	49	IFNAR1	30	HLA-DRB1	17	CASP6	7	FCER2
100	CSF2	49	APOE	29	UBE2I	17	PTBP1	7	CLEC12A
97	ICAM1	49	NOS3	29	RB1	17	PIK3CG	7	VAPA
97	STAT1	48	TRAF3	29	PSMC6	17	PLA2G4A	7	CLEC4M
96	IL1B	48	MX1	29	BCL2	16	BID	7	RAPGEF3
96	CCL2	48	MCL1	29	EIF2AK3	16	ERN1	7	SLC3A2
87	IL17A	47	TFRC	29	BST2	16	MBL2	6	PHB2
85	RELA	47	CXCL2	29	PYCARD	16	FBL	6	PRKRA
82	MAPK14	47	FCGR2A	29	ADAM17	16	UBD	6	SPINT1
80	CCL5	46	CREBBP	29	TOLLIP	16	DEFB4A	6	MYOM2
78	CXCL10	46	IL1A	28	EIF4E	16	PPIF	6	CTRL
77	IL18	46	CCL3	27	HNRNPA1	15	CCND3	5	SRPK1
76	FOS	46	SERPINE1	27	BAX	15	PIK3CD	5	ATP1A1
76	IRF1	45	CXCR3	27	APOA1	15	ARF1	5	APOD
76	TLR10	45	DDIT3	27	HLA-C	15	DROSHA	4	GBF1
75	CASP8	45	GPT	27	SMAD7	15	BCL2L2	4	CEACAM3
75	IRF3	43	IFNA1	27	HAVCR2	15	ACE2	4	LMAN2
75	PTGS2	42	CD4	26	ATF2	15	CAMK2D	3	HPN
74	NFKB1	41	HSP90B1	26	HLA-B	15	CARD9	3	LCN1
73	TRAF6	41	CBL	26	IFNL1	14	ITGA5	3	NMRAL1
72	FGF2	41	CXCL11	26	CD14	14	PHB	3	CST5
71	IL13	41	HSPB1	25	PRKCA	14	HFE	3	SGTA
69	ANXA5	40	PIK3R1	25	EZR	14	MAP1LC3A	3	TMPRSS11D
68	BCL2L1	40	TBK1	25	ANPEP	14	SPTAN1	2	MASP2
68	CD40LG	40	SOD1	24	PIK3C2A	14	CEACAM1	2	ATP6V1G1
68	CRP	40	PARP1	23	ICAM3	14	PTGS1	2	ZCRB1
68	CD34	39	RELB	23	GZMA	14	SCARB1	2	PCSK7
66	IL5	38	MAVS	22	CD3D	14	APOBEC3G	1	PGLS
65	IFNB1	38	ACE	22	PIK3R2	14	CEACAM5	1	MPP5
63	DDX58	37	CCL11	22	CD3G	13	BAD	1	TMPRSS13
63	STAT6	37	CCR1	22	IFITM3	13	RUNX1	1	MCRS1
62	JAK1	36	GRB2	22	CP	12	KPNA2	1	CHKB
61	CCR5	36	CTSB	22	RNASE3	12	MAPKAPK2	1	HELLS
61	SOCS3	35	ITGAL	21	EIF2S1	12	VHL	1	NUDT2
59	CREB1	35	CANX	21	IFITM1	12	DDX1	1	CLEC4G
58	HMOX1	35	CD209	21	CD81	12	SH2D3A	1	TMPRSS11A
57	ISG15	35	DPP4	21	TTR	11	ICAM2		
57	PPARG	34	CCR3	20	KPNB1	11	NMI		
56	CAT	34	TRIM25	20	POU5F1	11	TMPRSS2		
55	EGR1	33	CD3E	20	CST3	10	F8		

### 3.3 PPI network of targets for XBJ injection in treating COVID-19

**Figure 2.**
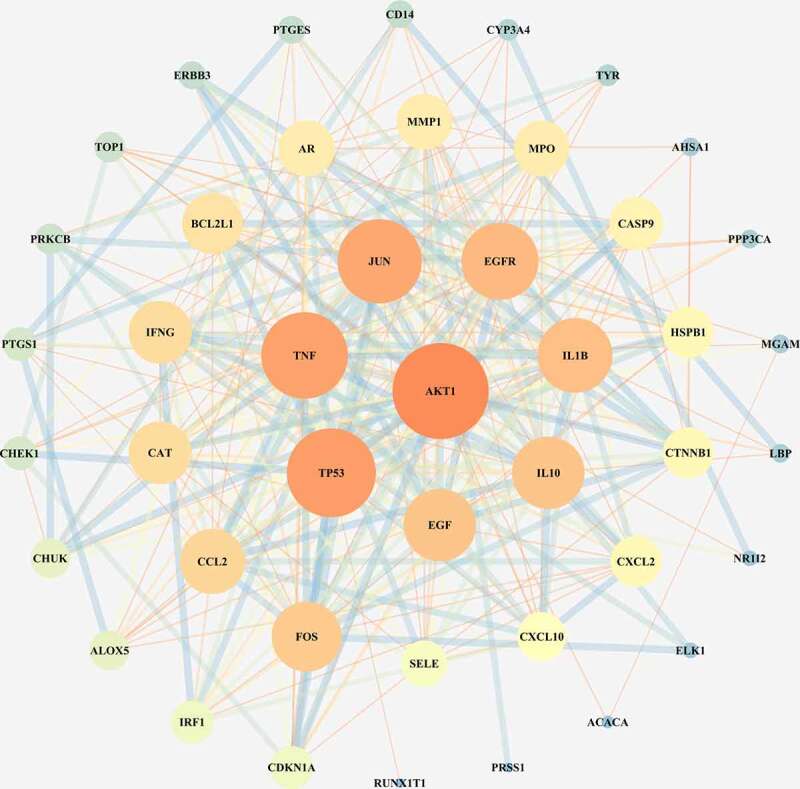
Junction targets of the XBJ injection and COVID-19. The darkness of the color and size of the circle are positively correlated with the role of the target in the network

The 46 junction targets of the Xuebijing injection and COVID-19 were imported into the STRING database to construct a PPI network (average node degree was 13.1, PPI enrichment p-value < 1.0 × 10^−16^, avg. local clustering coefficient was 0.675). The PPI network was then optimized using the Cytoscape software (see Figure 2), and the core target AKT1 was identified according to Degree ≥ 34. Secondary targets were also screened according to 24 ≤ Degree < 34, and included TP53, TNF, JUN, EGFR, IL1B, IL10, and EGF.

### 3.4 Analysis of the core target AKT1 from the XBJ injection when treating COVID-19

#### 3.4.1 GO enrichment analysis

**Figure 3.**
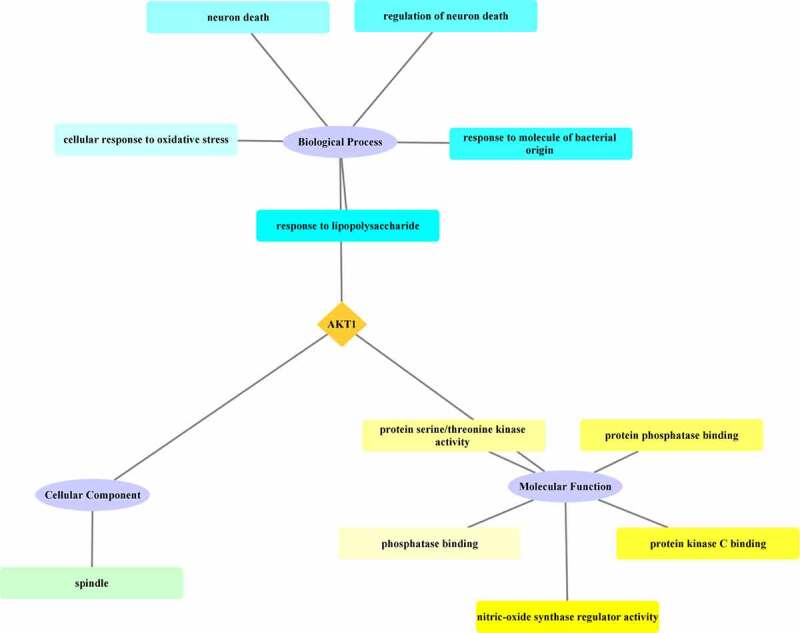
Invovlement of the significant GO enrichments as determined from the core target AKT1. The darkness of the color was inversely proportional to the P value

GO enrichment analysis of the core target AKT1 from the junction targets mapped for the XBJ injection and COVID-19 was conducted using the clusterProfiler package and Cytoscape 3.7.2 was used to plot the results. According to the results of the GO enrichment analysis, the main biological processes in which AKT1 was involved included response to lipopolysaccharide, response to molecule of bacterial origin, regulation of neuron death, neuron death, and cellular response to oxidative stress. The cellular component was spindle. The molecular functions in which AKT1 was found to be involved included nitric-oxide synthase regulator activity, protein kinase C binding, protein phosphatase binding, protein serine/threonine kinase activity, and phosphatase binding. See Figure 3.

#### 3.4.2 KEGG pathway analysis

**Figure 4.**
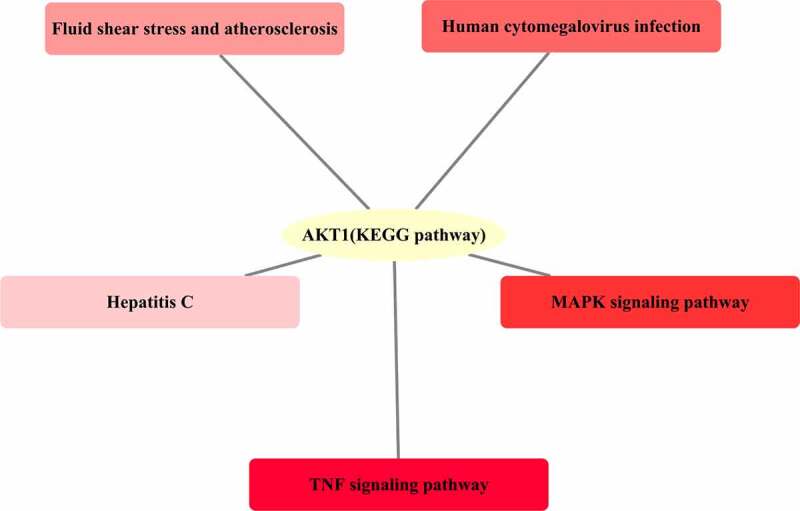
Involvement of the significant KEGG pathways as determined by the core target AKT1. The darkness of the color was inversely proportional to the P value

The KEGG pathway analysis of the 46 junction targets mapped to the XBJ injection and COVID-19 was conducted using the clusterProfiler package and Cytoscape 3.7.2 was used to plot the results. The results showed that the KEGG pathways in which AKT1 was involved included the TNF signaling pathway, MAPK signaling pathway, human cytomegalovirus infection, fluid shear stress and atherosclerosis, and hepatitis C (see Figure 4).

#### 3.4.3 Active ingredients that are connected with the core target AKT1

**Figure 5.**
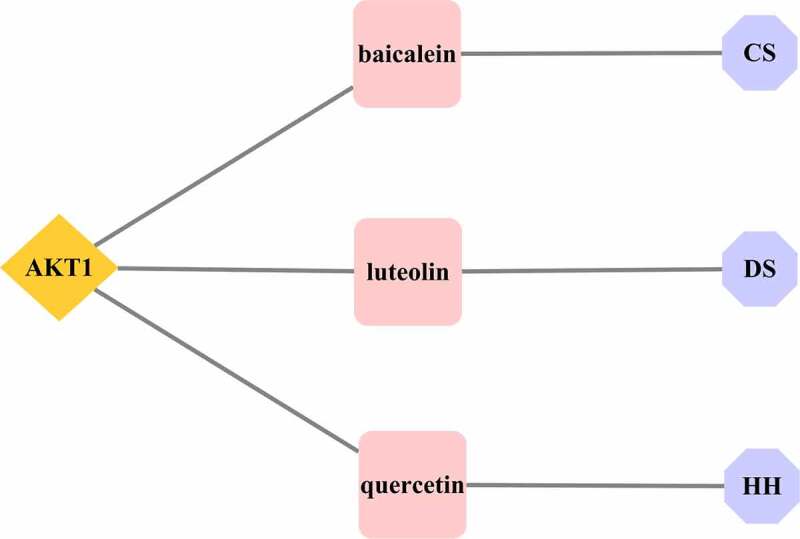
Active ingredients of the core target AKT1

The active ingredients of the XBJ injection, included baicalein (Paeoniae Radix Rubra), luteolin (Salviae Miltiorrhizae Radix et Rhizoma), and quercetin (Carthami Flos), and they were all connected with AKT1, suggesting that these three active ingredients may be effective for XBJ injection in the prevention and treatment of COVID-19 (see Figure 5).

3.5 Verification of molecular docking for the XBJ injection when treating COVID-19 by the Results of Network Pharmacology

**Figure 6.**
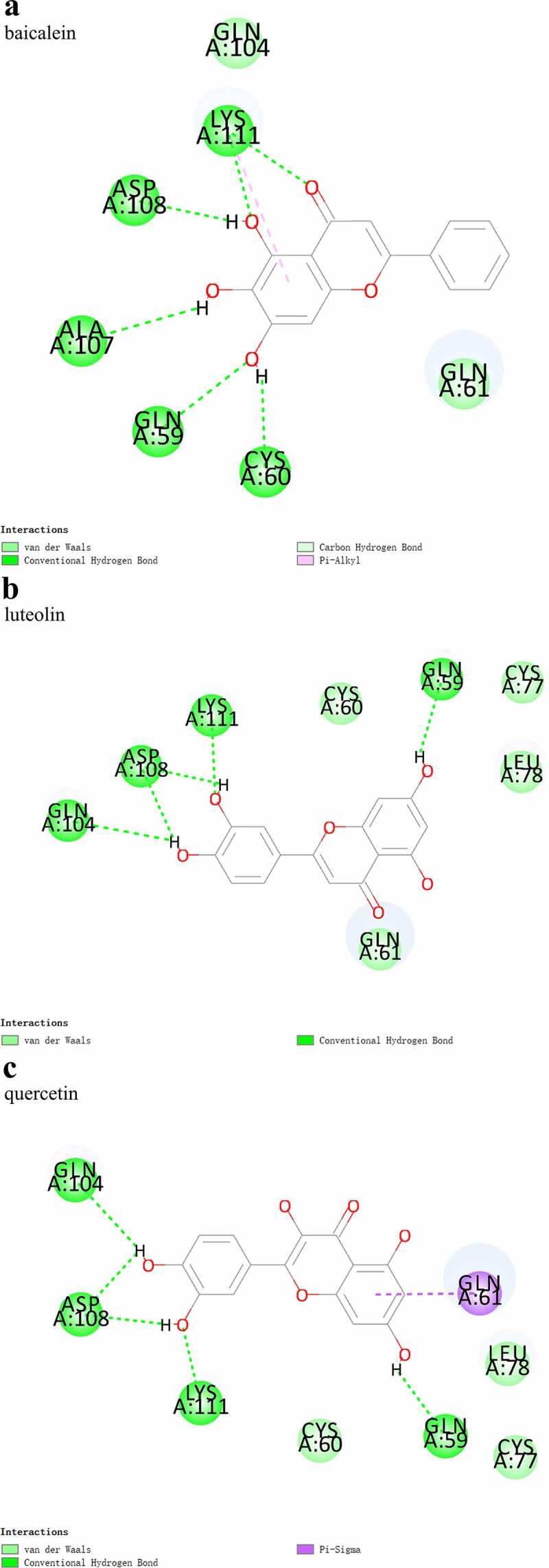
Binding models between AKT1 and baicalein (a), luteolin (b), and quercetin (c)

The activity of the three active ingredients obtained through the network pharmacology analysis were validated using protein-small molecule docking.The results showed stable binding between the three active ingredients (baicalein, luteolin, and quercetin) and AKT1, with binding energies of −25.5854 kcal·mol^−1^, −31.5575 kcal·mol^−1^, and −31.53 kcal·mol^−1^, respectively. The binding models are shown in Figure 6a−6c.

## 4. Discussion

According to the *Diagnosis and Treatment Protocol for COVID-19 (Trial Version 8*), critically ill patients are more likely to develop dyspnea and/or hypoxemia within a week, and severe patients, like those with SARS and MERS, may suffer from a cytokine storm (CS), which can rapidly develop into acute respiratory distress syndrome, septic shock, and multiple organ failure. The XBJ injection has been shown in previous studies to be effective at improving end-point outcomes for severe community-acquired pneumonia (CAP) and reduced the 28-day mortality in septic patients [12].

In this study, 38 non-repeated active ingredients and 206 non-repeated potential targets of the XBJ injection, 277 potential targets of COVID-19, and 46 junction targets were screened using network pharmacology analysis, and the core target from the junction targets was identified to be AKT1. AKT1 is a member of the AKT kinase family that regulates metabolism, proliferation, cell survival, growth, and angiogenesis through a series of downstream substrates. AKT regulates the metabolism of fat, amino acids, and other substances by activating AS160 and PFKFB2, and also induces the TSC1/TSC2 complex and mTORC signal transduction after the stimulation of inflammatory factors, so as to regulate the growth of the endothelium, restore the endothelial barrier function, and promote proliferation of the lung fibroblasts [13–15]. A previous study has shown that overexpressed constitutively active AKT1 can promote viral protein synthesis [16]. Furthermore, activation of the PI3K/AKT pathway is indispensable for coxsackievirus B3 infection. Dominant negative mutant of AKT1 can significantly dampen viral RNA expression and further reduce viral capsid protein expression and viral release [17]. The replication of another coronavirus, Middle East respiratory syndrome coronavirus, was remarkably inhibited when kinase inhibitors that target PI3K/AKT were adminstered [18]. Collectively, AKT1 could be an ideal target with a broad-spectrum antiviral effect. These results suggest that the XBJ injection is an effective control and treatment for COVID-19, probably due to its regulation of AKT1.

According to the results of the GO enrichment analysis, the response to lipopolysaccharide (LPS) was the most significant biological process that AKT1, the core target of junction targets to the XBJ injection and COVID-19, was involved. LPS is a major component in the cell walls of Gram-negative bacteria. It has been reported that LPS can damage alveolar epithelial cells and capillary endothelium cells, resulting in changes to the intercellular space and permeability [19,20]. AKT could regulate the NF-κB signal transduction through phosphorylated IKKα and Tpl2 in order to ameliorate lipopolysaccharide-induced alveolar blood stasis and abnormal fibrinolysis in mice [21]. It has been suggested that XBJ injection may inhibit response to lipopolysaccharide by regulating AKT1 expression, thus alleviating lung injury caused by LPS. The results of this study showed that the most significant molecular function of AKT1 is the nitric-oxide synthase regulator activity. Nitric-oxide synthase (NOS) is an isozyme that is reportedly associated with pneumonia and pulmonary fibrosis [22]. These results suggest that XBJ injection may regulate NOS activity through AKT1, which may help to prevent and treat COVID-19.

According to the results of the KEGG pathway analysis, the TNF signaling pathway is the most significant pathway in which the core target AKT1, that is the core target of junction targets to XBJ injection and COVID-19, was involved. Tumor necrosis factors (TNF) include TNF-α and TNF-β which can promote the expression of proinflammatory factors and participate in systemic inflammatory responses, while it can also produce an anti-infection effect and prevent early viral proteins from being synthesized to inhibit viral replication and kill infected cells. Therefore, the XBJ injection can regulate the TNF signal pathway through AKT1, so that it has anti-inflammation and anti-virual effects, and thus the potential to reduce the inflammation caused by COVID-19.

The protein-small molecule docking analysis revealed that all three active ingredients (baicalein, luteolin, and quercetin) had perfect CDOCKER Interaction Energy with AKT1 (1UNP), with the highest found in luteolin. This inferred that luteolin was the most important active ingredient contributing to the therapeutic effects of the XBJ injection on COVID-19. The analysis of the binding model showed that there were conventional hydrogen bonds between the luteolin and Gln104, Asp108, Lys111, and Gln59 of the AKT1 and van der Waals between luteolin and Cys60, Gln61, Cys77, and Leu78 of AKT1. It has been reported that luteolin can mitigate airway inflammation by regulating the body’s antioxidative stress response and the COX-2 signaling pathway. Luteolin is also shown to relieve myocardial ischemic injury by modulating the mitogen-activated protein kinase (MAPK)/NF-κB pathway and suppressing enterovirus type 71 infections by inhibiting cell apoptosis and inflammatory factor secretion, demonstrating its antiviral properties. Additionally, luteolin and quercetin are found to induce cell apoptosis by inhibiting the phosphorylation and activation of AKT [23–25]. Despite all these findings, it has not been reported whether these three active ingredients relieve COVID-19 symptoms through the direct regulation of AKT1. In this study, molecular docking was implemented to further explore the mechanisms of action of the three active ingredients in treating COVID-19. Furthermore, considering that baicalein, luteolin, and quercetin are found in many herbal components, these active ingredients may not be unique to the XBJ injection, indicating the possibility of discovering antiviral TCM combinations in other ancient prescriptions containing baicalein, luteolin, and quercetin.

In this study, potential targets were screened, a PPI network was constructed, and enrichment analysis was conducted to find potential pathways. The XBJ injection was shown to produce synergistic therapeutic effects on COVID-19 with ‘multi- active ingredients, multi-target and multi-pathway’, and molecular docking was performed to verify such results. However, the present study has some limitations: 1) The study results are based on existing data available in the given database, and differences in the results may arise if different databases were used. Therefore, future studies should focus on multidatabase analysis and databases that are updated regularly. 2) The biological network in this study was constructed using qualitative data, but TCM prescriptions usually have a complex composition, as well as intricate interactions, making it extremely difficult to perform an analysis based on the quantitative data. Consequently, the reliability of the conclusions from this study need to be further verified. Therefore, in vivo and in vitro experiments should be conducted to further examine the molecular mechanisms of the XBJ injection for the treatment of COVID-19.

## 5. Conclusion

In conclusion, according to the network pharmacology and molecular docking analyses, the predicted active ingredients in the XBJ injection include baicalein, luteolin, and quercetin. The XBJ injection may inhibit the lipopolysaccharide-mediated inflammatory response, modulate NOS activity, and regulate the TNF signaling pathway by affecting the function of the AKT1, thereby ultimately suppressing the excessive inflammatory response associated with COVID-19. Due to the limitations of the computational methods for chemistry and biology, the results need to be verified with follow-up experiments to provide a basis for the treatment of COVID-19 with TCM.

## Figures and Tables

**Table:** 

Abbreviation	Full name
3D	3-dimension
ADRB2	adrenoceptor beta 2
AKT1	AKT serine/threonine kinase 1
ARDS	acute respiratory distress syndrome
AS160	TBC1 domain family member 4
Asp	aspartic acid
BP	biological process
CAP	community-acquired pneumonia
CC	cellular component
CHARMm	Chemistry at HARvard Macromolecular Mechanics
COVID-19	Corona Virus Disease 2019
COX-2	prostaglandin-endoperoxide synthase 2
CS	Cytokine Storm
Cys	cysteine
DL	Drug-Likeness
DS	Discovery Studio 2019 Client
EGF	epidermal growth factor
EGFR	epidermal growth factor receptor
FDA	Food and Drug Administration
GAPDH	glyceraldehyde-3-phosphate dehydrogenase
Gln	glutamine
GO	Gene Ontology
HCoVs	human coronaviruses
IKKα	Inhibitory Kappa B Kinase α
IL10	Interleukin 10
IL1B	Interleukin 1 beta
IL6	Interleukin 6
JUN	Jun proto-oncogene, AP-1 transcription factor subunit
KCNH2	potassium voltage-gated channel subfamily H member 2
KEGG	Kyoto Encyclopedia of Genes and Genomes
Leu	Leucine
LPS	Lipopolysaccharide
Lys	lysine
MAPK	mitogen-activated protein kinase
MERS	Middle East respiratory syndrome
MF	molecular function
MOF	multiple organ failure
mTORC	mammalian target of rapamycin complex
NCOA1	nuclear receptor coactivator 1
NCOA2	nuclear receptor coactivator 2
NF-κB	nuclear transcription factor-κB
NOS	nitric-oxide synthase
NR3C2	nuclear receptor subfamily 3 group C member 2
OB	Oral Bioavailability
PDB	Protein Data Bank
PDE3A	phosphodiesterase 3A
PFKFB2	6-phosphofructo-2-kinase/fructose-2,6-biphosphatase 2
PGR	progesterone receptor
PHEIC	Public Health Emergency of International Concern
PI3K	phosphatidylinositol-4,5-bisphosphate 3-kinase catalytic subunit alpha
PPI	protein-protein interaction
PTGS1	prostaglandin-endoperoxide synthase 1
PTGS2	prostaglandin-endoperoxide synthase 2
RCSB	Research Collaboratory for Structural Bioinformatics
RNA	Ribonucleic Acid
RXRA	retinoid X receptor alpha
SARS	severe acute respiratory syndrome
SARS-CoV-2	severe acute respiratory syndrome coronavirus 2
SCN5A	sodium voltage-gated channel alpha subunit 5
SDF	Standard Delay Format
svg	Scalable Vector Graphics
TCM	Traditional Chinese medicines
TCMSP	Traditional Chinese Medicine Systems Pharmacology Database and Analysis Platform
TNF	Tumor Necrosis Factors
TP53	Tumor Protein p53
Tpl2	mitogen-activated protein kinase kinase kinase 8
TSC1	TSC complex subunit 1
TSC2	TSC complex subunit 2
TSV	Tab-separated values
UniProt	Universal Protein Resource
WHO	World Health Organization
XBJ	Xuebijing
XFZYD	XueFuZhuYu Decoction

## Data Availability

Publicly available datasets were analyzed in this study. This data can be found here: [GeneCards] at [https://www.genecards.org/], [Traditional Chinese Medicine Systems Pharmacology Database (TCMSP)] at [http://tcmspw.com/tcmsp.php], [UniProt] at [https://www.uniprot.org/], [STRING] at [https://STRING-db.org/cgi/input.pl], [Pubchem] at [https://pubchem.ncbi.nlm.nih.gov/], [RCSB Protein Data Bank] at [http://www.rcsb.org/].

## Highlight

(1) AKT1 was identified as the core target of Xuebijing injection used to treat COVID-19.

(2) Xuebijing injection may inhibit lipopolysaccharide-mediated inflammatory response.

(3) Xuebijing injection may regulate nitric-oxide synthase activity and the TNF signal pathway.

(4) Baicalein, luteolin, and quercetin may be the active ingredients of Xuebijing injection connected with AKT1.
